# Supplementary material for: Oxidative Stress and Deregulated DNA Damage Response Network in Lung Cancer Patients
Source: Biomedicines. 2022 May 26;10(6):1248. doi: 10.3390/biomedicines10061248 (PMC9219789; doi:10.3390/biomedicines10061248)
Supplement: Supplementary file 1 [file biomedicines-10-01248-s001.zip › biomedicines-1709887-supplementary/biomedicines-1709887-supplementary.pdf]

# Supplementary Materials

## Table of Contents


- Table S1. Genes demonstrating at least 2-fold difference in the transcription activity between lung cancer patients and healthy controls
- Figure S1. ATM Signaling pathway
- Figure S2. Role of BRCA1 in DNA Damage Response
- Figure S3. Base Excision Repair Pathway
- Figure S4. Role of CHK proteins in Cell Cycle Checkpoint Control
- Figure S5. Cell Cycle: G2/M DNA Damage Checkpoint Regulation
- Figure S6. DNA Double-Strand Break Repair by Non-Homologous End Joining
- Figure S7. Cyclins and Cell Cycle Regulation
- Figure S8. p53 Signaling
- Figure S9. Color and shape legends useful for IPA® pathway/network representation

**Table S1. Genes demonstrating at least 2-fold difference in the transcription activity between lung cancer patients and healthy controls**

| Ingenuity Canonical Pathways | Molecules |
|---|---|
| ATM Signaling | ATM↓, CDC25A↑, CHEK1↑, MDC1↑, RAD51↑, RAD9A↑, RBBP8↑, RNF168↑, TP53↓, TP53BP1↑ |
| Role of BRCA1 in DNA Damage Response | ATM↓, CHEK1↑, MDC1↑, MLH1↓, MSH2↓, RAD51↑, RBBP8↑ TP53↓ |
| BER (Base Excision Repair) Pathway | APEX1↓, MPG↓, OGG1↓, PARP1↑, PNKP↑, TP53↓ |
| Role of CHK Proteins in Cell Cycle Checkpoint Control | ATM↓, CDC25A↑, CHEK1↑, MDC1↑, RAD9A↑, TP53↓ |
| Cell Cycle: G2/M DNA Damage Checkpoint Regulation | ATM↓, CDK7↑, CHEK1↑, PRKDC↑, TP53↓ |
| DNA Double-Strand Break Repair by Non-Homologous End Joining | ATM↓, PARP1↑, PRKDC↑ |
| Cyclins and Cell Cycle Regulation | ATM↓, CHEK1↑, MDC1↑, MLH1↓, MSH2↓, RAD51↑, RBBP8↑ TP53↓ |
| p53 Signaling | ATM↓, CHEK1↑, PRKDC↑, TP53↓ |
| Mismatch Repair in Eukaryotes | EXO1↑, MLH1↓, MSH2↓ |
| DNA Double-Strand Break Repair by Homologous Recombination | ATM↓, RAD51↑ |
| NER (Nucleotide Excision Repair, Enhanced Pathway) | CDK7↑, DDB1↑, DDB2↑ |

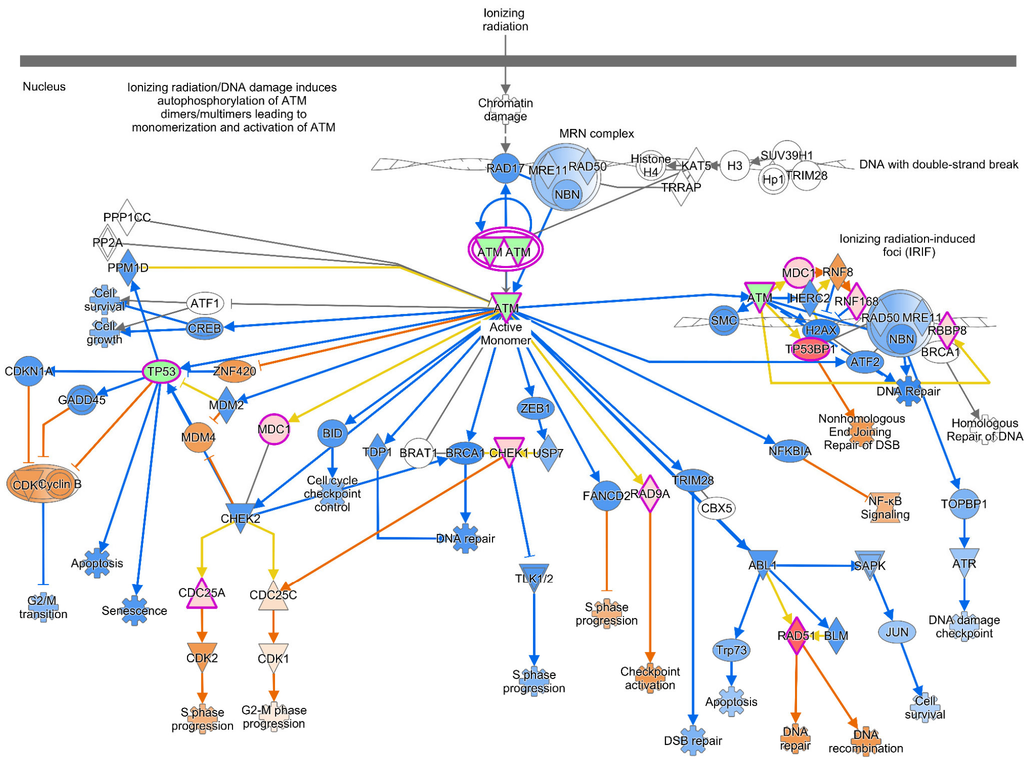


**Figure S1**. ATM Signaling pathway. Canonical pathways related to DNA damage response and repair from IPA® presenting statistically significant (*P*<0.05) enrichment: ATM Signaling (*P*=3.03E-18). Color and shape legends are presented in Figure S9.

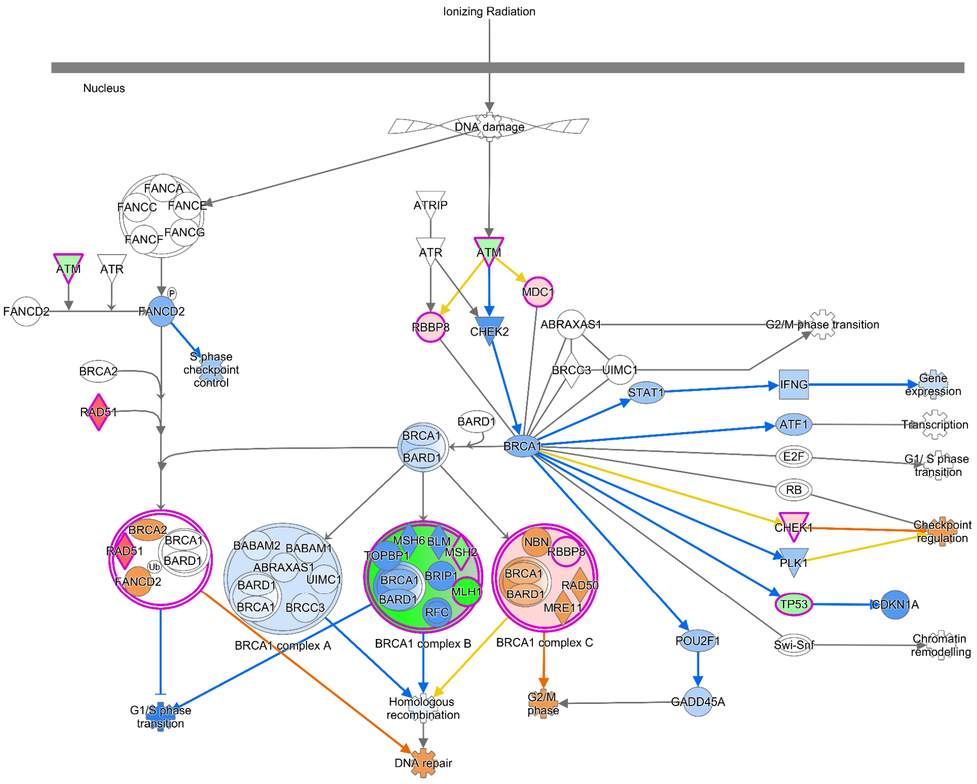


**Figure S2**. Role of BRCA1 in DNA Damage Response. Canonical pathways related to DNA damage response and repair from IPA® presenting statistically significant (*P*<0.05) enrichment: Role of BRCA1 in DNA Damage Response (*P*=1.18E-14). Color and shape legends are presented in Figure S9.

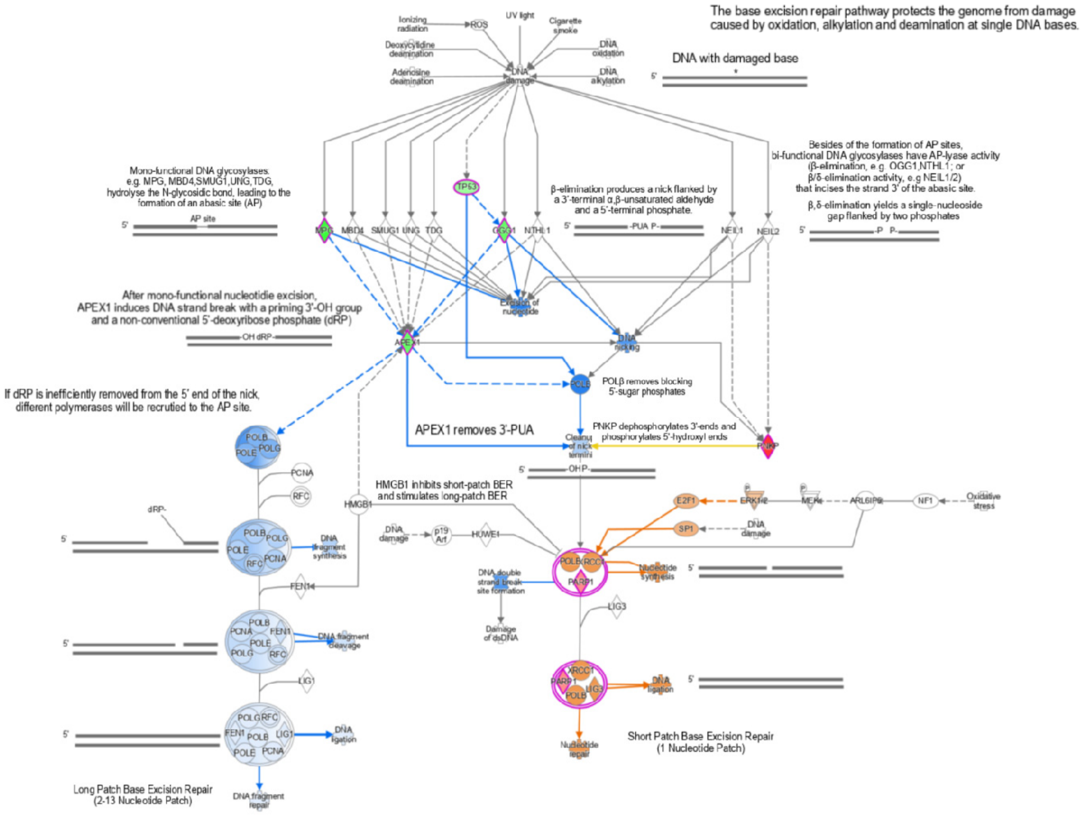


**Figure S3.** Base Excision Repair Pathway. Canonical pathways related to DNA damage response and repair from IPA® presenting statistically significant ($P$<0.05) enrichment: BER pathway ($P$=4.84E-12). Color and shape legends are presented in Figure S9.

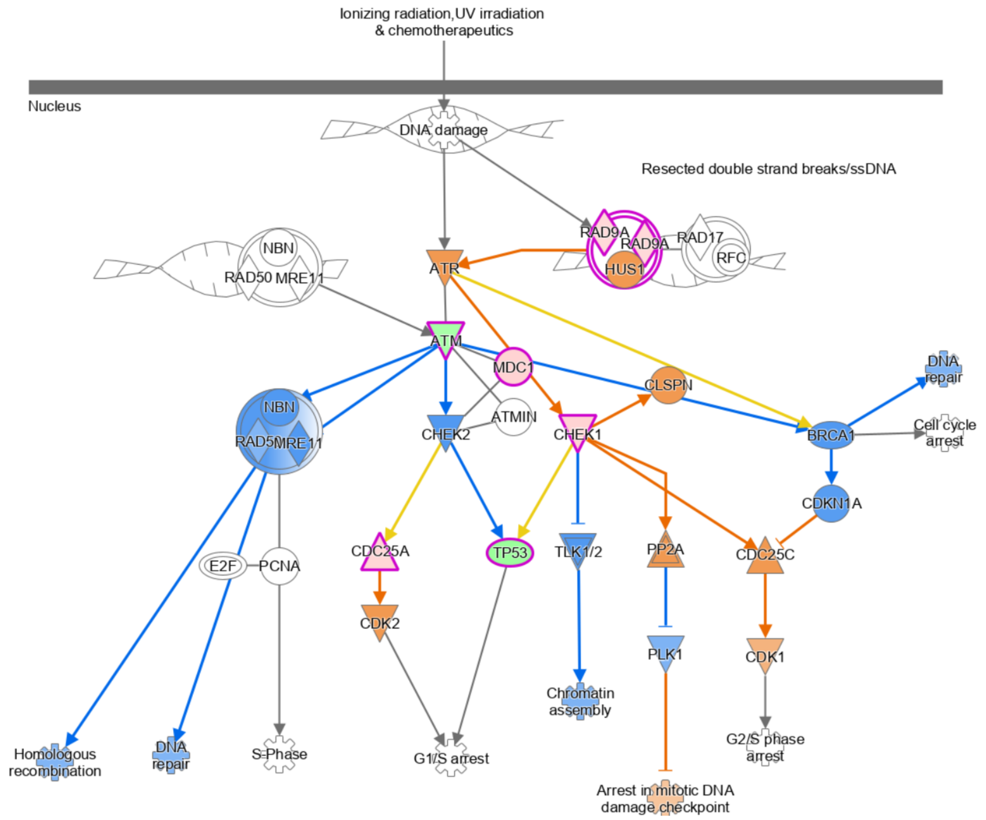


**Figure S4.** Role of CHK proteins in Cell Cycle Checkpoint Control. Canonical pathways related to DNA damage response and repair from IPA® presenting statistically significant ($P$<0.05) enrichment: Role of CHK Proteins in Cell Cycle Checkpoint Control ($P$=2.46E-11). Color and shape legends are presented in Figure S9.

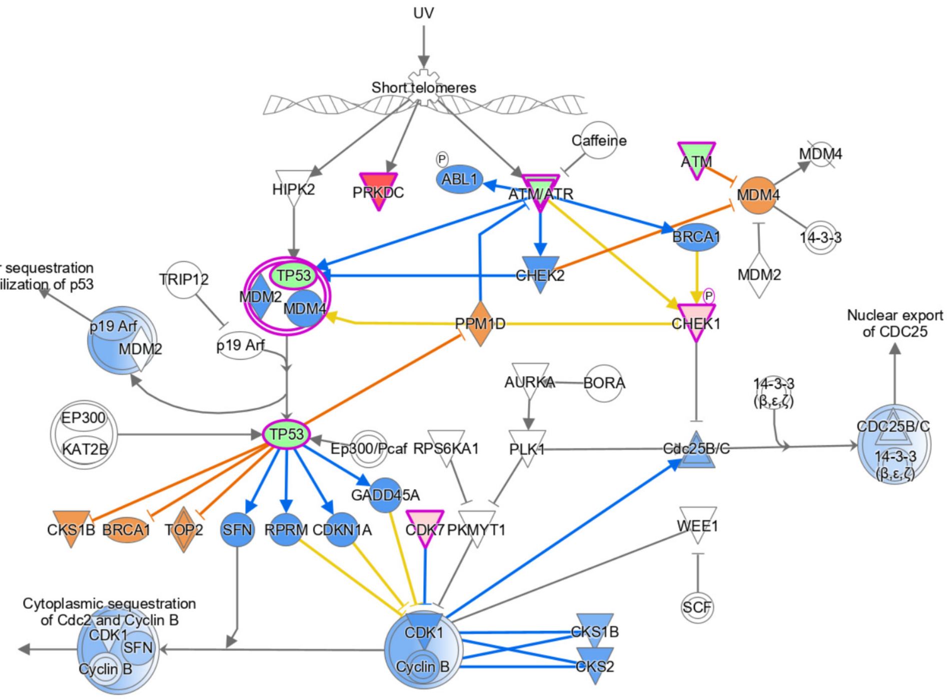


**Figure S5.** Cell Cycle: G2/M DNA Damage Checkpoint Regulation. Canonical pathways related to DNA damage response and repair from IPA® presenting statistically significant ($P$<0.05) enrichment: Cell Cycle: G2/M DNA Damage Checkpoint Regulation ($P$=1.72E-09). Color and shape legends are presented in Figure S9.

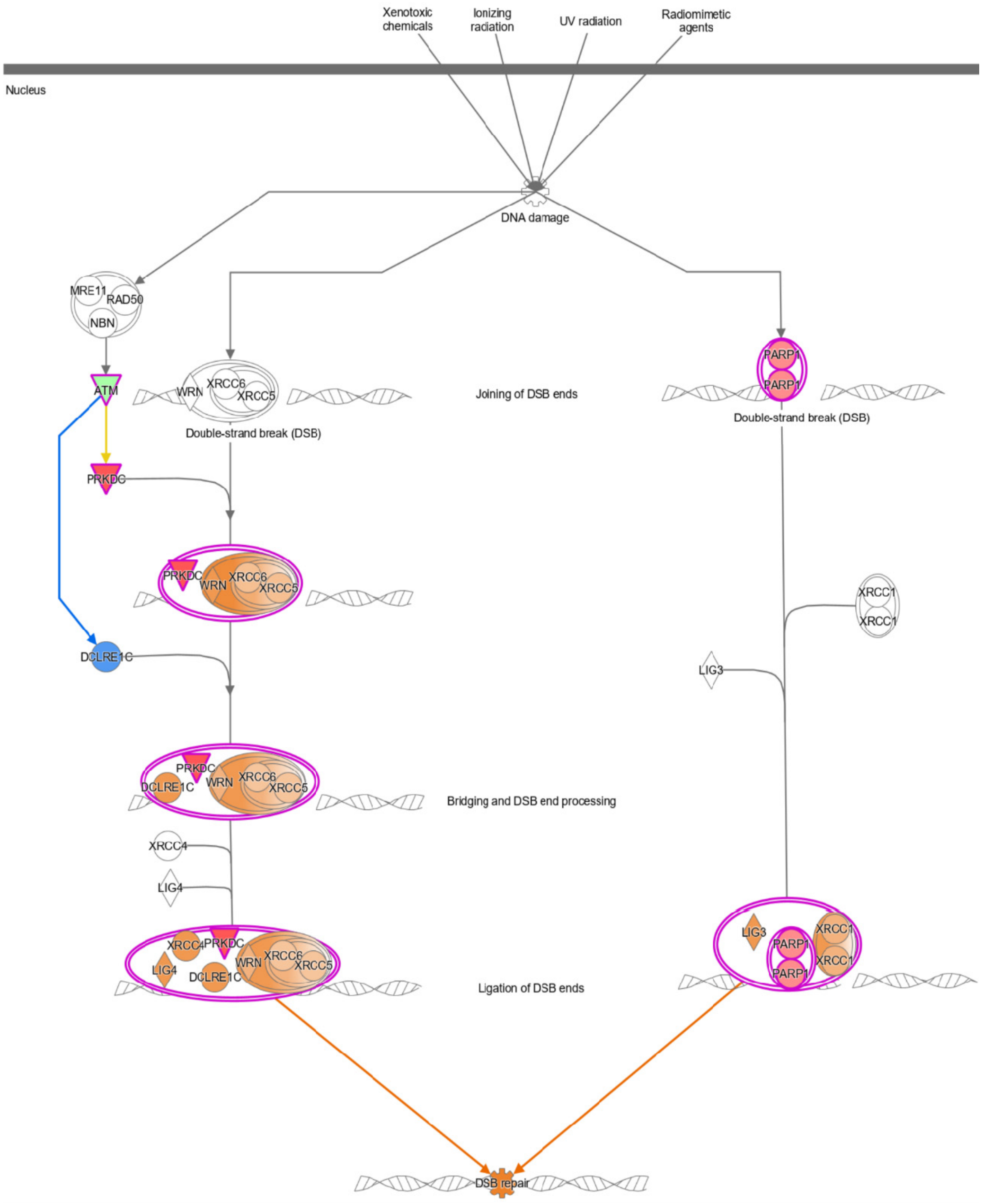


**Figure S6.** DNA Double-Strand Break Repair by Non-Homologous End Joining. Canonical pathways related to DNA damage response and repair from IPA® presenting statistically significant ($P$<0.05) enrichment: DNA Double-Strand Break Repair by Non-Homologous End Joining ($P$=3.7E-07). Color and shape legends are presented in Figure S9.

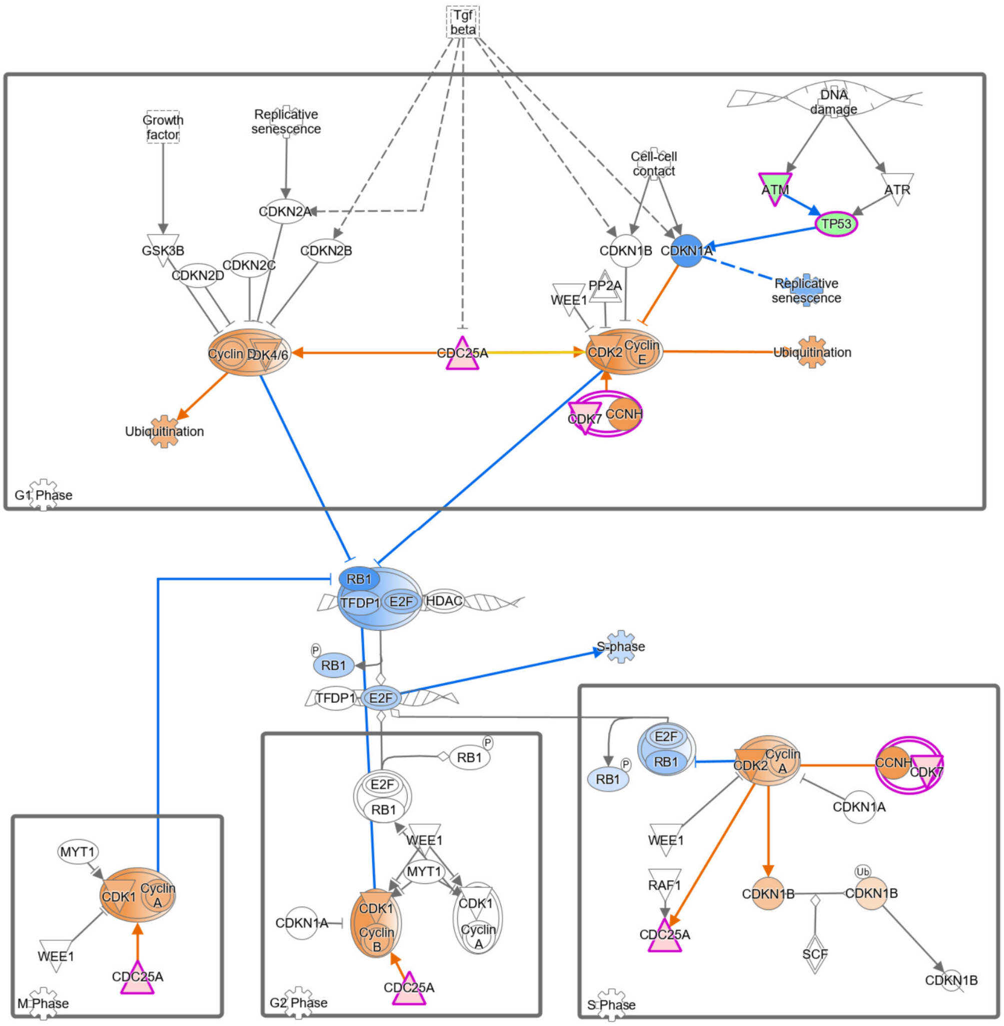


**Figure S7.** Cyclins and Cell Cycle Regulation. Canonical pathways related to DNA damage response and repair from IPA® presenting statistically significant (*P*<0.05) enrichment: Cyclins and Cell Cycle Regulation (*P*=1.73E-06). Color and shape legends are presented in Figure S9.

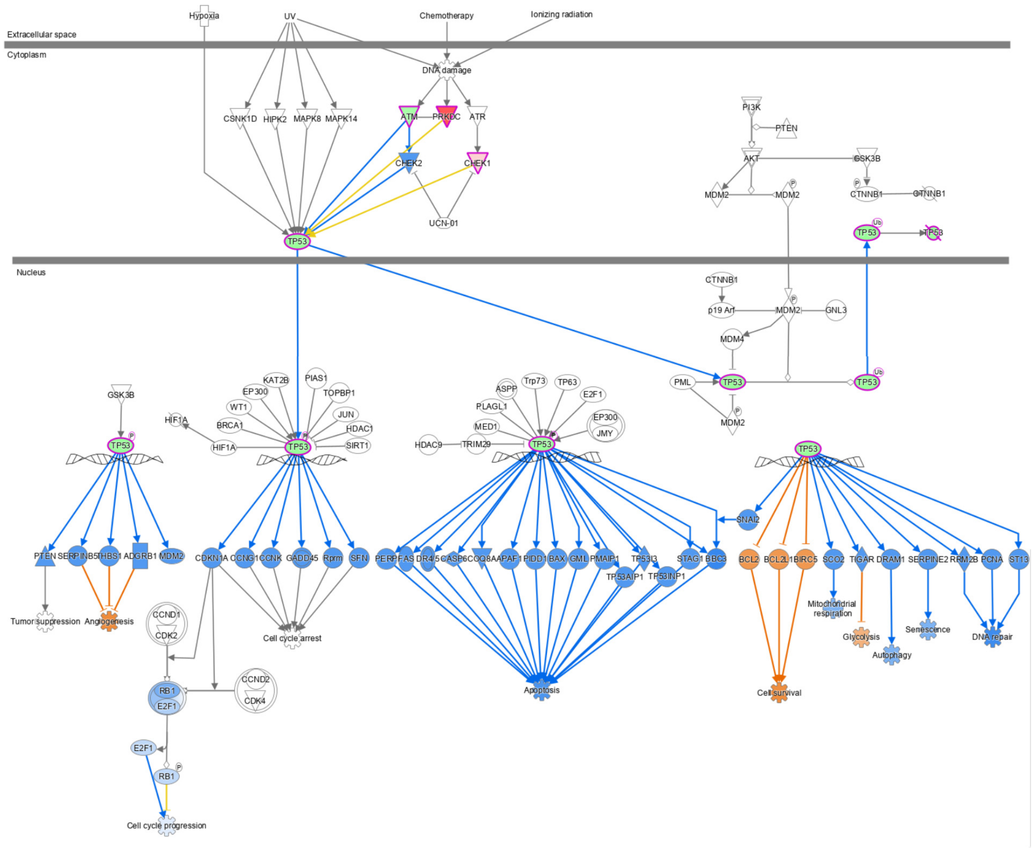


**Figure S8**. p53 Signaling. Canonical pathways related to DNA damage response and repair from IPA® presenting statistically significant ($P$<0.05) enrichment: p53 Signaling ($P$=3.2E-06). Color and shape legends are presented in Figure S9.

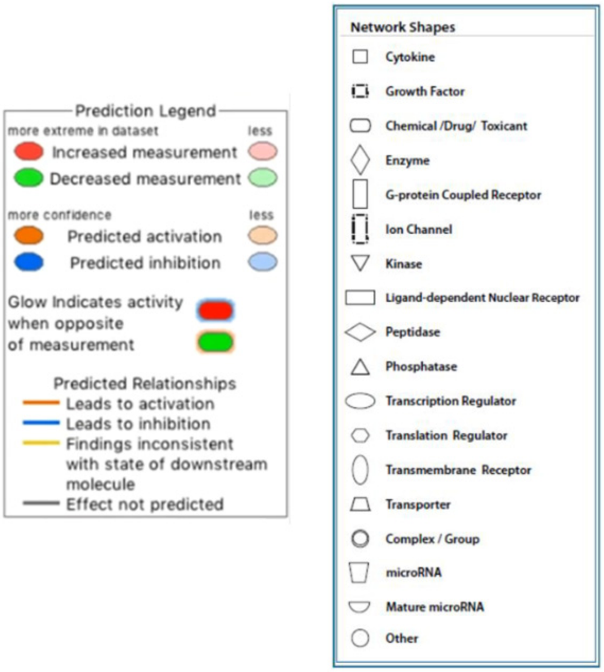


**Figure S9**. Color and shape legends useful for IPA® pathway/network representation.
